# Improving child health promotion practices in multiple sectors – outcomes of the Swedish Salut Programme

**DOI:** 10.1186/1471-2458-12-920

**Published:** 2012-10-30

**Authors:** Kristina Edvardsson, Anneli Ivarsson, Rickard Garvare, Eva Eurenius, Marie Lindkvist, Ingrid Mogren, Rhonda Small, Monica E Nyström

**Affiliations:** 1 Department of Public Health and Clinical Medicine, Epidemiology and Global Health, Umeå University, SE 901 87, Umeå, Sweden; 2 Mother and Child Health Research, La Trobe University, Melbourne, Vic, 3000, Australia; 3 Division of Quality Management, Luleå University of Technology, SE 971 87, Luleå, Sweden; 4 Department of Statistics, Umeå University, SE 90187, Umeå, Sweden; 5 Department of Clinical Sciences, Obstetrics and Gynecology, Umeå University, SE 901 87, Umeå, Sweden; 6 Department of Learning, Informatics, Management and Ethics, Medical Management Centre, Karolinska Institutet, SE 171 77, Stockholm, Sweden

**Keywords:** Antenatal care, Change barriers, Change facilitators, Child health care, Complex interventions, Dental health services, Health promotion, Implementation, Prevention, Primary health care

## Abstract

**Background:**

To improve health in the population, public health interventions must be successfully implemented within organisations, requiring behaviour change in health service providers as well as in the target population group. Such behavioural change is seldom easily achieved. The purpose of this study was to examine the outcomes of a child health promotion programme (The Salut Programme) on professionals’ self-reported health promotion practices, *and* to investigate perceived facilitators and barriers for programme implementation.

**Methods:**

A before-and-after design was used to measure programme outcomes, and qualitative data on implementation facilitators and barriers were collected on two occasions during the implementation process. The sample included professionals in antenatal care, child health care, dental services and open pre-schools (n=144 pre-implementation) in 13 out of 15 municipalities in a Swedish county. Response rates ranged between 81% and 96% at the four measurement points.

**Results:**

Self-reported health promotion practices and collaboration were improved in all sectors at follow up. Significant changes included: 1) an increase in the extent to which midwives in antenatal care raised issues related to men’s violence against women, 2) an increase in the extent to which several lifestyle topics were raised with parents/clients in child health care and dental services, 3) an increased use of motivational interviewing (MI) and separate ‘fathers visits’ in child health care 4) improvements in the supply of healthy snacks and beverages in open pre-schools and 5) increased collaboration between sectors. Main facilitators for programme implementation included cross-sectoral collaboration and sector-specific work manuals/questionnaires for use as support in everyday practice. Main barriers included high workload, and shortage of time and staff.

**Conclusion:**

This multisectoral programme for health promotion, based on sector-specific intervention packages developed and tested by end users, and introduced via interactive multisectoral seminars, shows potential for improving health promotion practices and collaboration across sectors. Consideration of the key facilitators and barriers for programme implementation as highlighted in this study can inform future improvement efforts.

## Background

To improve health in the population, public health innovations must be successfully implemented within organisations, requiring behaviour change in health service providers as well as in the target population group. However, the literature to date indicates that such change is seldom easily achieved [1-5]. Numerous theories on behaviour change have been developed over the years [6], but there are no easy to follow recipes on how to achieve uptake of new public health innovations. This is because a number of different factors, often in interaction, will affect how innovations are disseminated, implemented and sustained in practice, and the influence of each factor is highly context-dependent [7]. Furthermore, features of the innovation itself have been shown to play a crucial role in individuals’ decisions to adopt or reject it. Innovations characterised by high relative advantage, compatibility, trialability, observability, and less complexity are more likely to be adopted than other innovations [8]. Innovations in health promotion and disease prevention are often characterised by low relative advantage, as outcomes are often revealed, if at all, after a long period of time. This partly explains why such innovations generally diffuse more slowly than innovations in other fields [9].

Adapting innovations to the local context is described as essential to improve uptake, a process that also involves an assessment of factors that can facilitate or impede change [10,11]. When planning for complex changes, Grol and Wensing emphasize the importance of considering factors at different levels, including features of the innovation; the professionals and patients; and the social, organisational, economic and political context [12]. Conceptual models, for example as developed by Greenhalgh et al., can also be used as tools to facilitate consideration of the situational complexity and the interaction of different factors [7]. Both quantitative and qualitative measures can contribute in illuminating factors that can facilitate or impede change. However, the use of qualitative methods in organisation and practice change trials has so far been relatively uncommon. This means that it is seldom known why an intervention was successful or not [13], hindering further learning, development and improvement [12,14]. To date, there are few implementation studies of complex public health innovations that span different organizations and involve other professions than physicians [7,15].

In 2005, the Västerbotten County Council in Sweden launched the Salut Programme with the aim to develop and strengthen health-promoting activities in health care, social services and school settings, and increase collaboration between these sectors [16,17]. The programme starts with the pregnant woman and her partner and continues to follow the child up to the age of 18 via age-adapted interventions grouped into modules. Interventions in the programme’s first two modules (from pregnancy to 18 months of age) were developed and tested in four pilot areas from 2005 to 2007 [16]. From 2008 to 2010 they were implemented in the rest of the county in three sequences. This study investigated the early outcomes of this attempt to improve health promotion practices and collaboration between antenatal care (ANC), child health care (CHC), dental services and open pre-schools. A more detailed description of involved sectors and the Salut Programme development can be found elsewhere [5,16,17]. The second (coastal areas) and third (inland areas) implementation sequences, involving 13 out of 15 municipalities in Västerbotten, were monitored for the purpose of this study.

The overall aim was to examine outcomes of the Salut Programme on professionals’ self-reported health promotion practices *and* to investigate perceived facilitators and barriers for programme implementation. The research questions were:

I. Are there significant changes in professionals’ self-reported health-promoting practices following programme implementation?

II. Are there significant changes in professionals’ self-reported collaboration between sectors following programme implementation?

III. What are the main facilitators and barriers for programme implementation as perceived by professionals, and do these differ between the time of initiation and completion, or between sectors?

## Methods

### Study design

A before-and-after design was used to measure programme outcomes, and qualitative data on implementation facilitators and barriers were collected on two occasions during the implementation process. Involved sectors were ANC, CHC, dental services and open pre-schools in 13 out of 15 municipalities in Västerbotten county (260,000 residents), Sweden.

### The Salut Programme implementation

The Salut Programme implementation process included four full-day seminars with lectures and intra- and inter-sectoral group discussions. Professionals were provided with sector specific work manuals for use in everyday practice and questionnaires for use as epidemiological surveillance tools. The questionnaires also served as a base for dialogues on health and lifestyle in ANC and dental services. Small scale testing of interventions between seminars was encouraged, and meetings conducted if necessary. The sector specific intervention packages have been described in more detail elsewhere [16]. The timeline for programme implementation, data collection, and the content of seminars are summarized in Table 1.

**Table 1:** **Timeline for programme implementation**, **data collection**, **and content of seminars 1**-**4 including approximate activity durtion**^**1**^

**Date**	**Coastal area**	**Date**	**Inland area**
**Feb 2009**	**Survey 1 (pre-implementation)**	**Aug 2009/Feb 2010**^**2**^	**Survey 1 (pre-implementation)**
**March 2009**	**1**. *Lectures* (*0*,*5 h*): Programme introduction and introduction of module I-II interventions.	**March 2010**	**1**. *Lectures* (*0*,*5 h*): Programme introduction and introduction of module I-II interventions.
	*Interactive lectures* (*2*,*5 h*): Children’s health of today and cross-sectoral collaborations.		*Interactive lectures* (*2 h*): Children’s health of today and cross-sectoral collaborations.
	*Group discussion* (*2*,*5 h*): Salut and my work.		*Group discussion* (*1*,*5 h*): Salut and my work.
**May 2009**	**2**. *Lectures* (*3 h*): Expectant parents’ health, parental support groups, parent–child attachment and fathers’ role.	**May 2010**	**2**. *Lectures* (*3 h*): Parent–child attachment, fathers’ role, intimate partner violence against women, social services’ role.
	*Group discussions* (*1*,*2 h*): Experience-sharing.		*Group discussions* (*1*,*75 h*): Parent child attachment/roles and summary of progress.
	**Survey 2(20 min)**		**Survey 2(20 min)**
**Feb 2010**	**3**. *Lectures* (*3*,*5 h*): Salut in the organisation, overweight in pregnancy, social services’ role and slideshows for parental meetings.	**Sept 2010**	**3**. *Lectures* (*3 h*): Motivational Interviewing, good family eating habits, and physical activity after pregnancy.
	*Group discussions* (*2 h*): Experience-sharing and what do we do next?		*Group discussions* (*1 h*): What has happened and what do we do next?
**April 2010**	**4**. *Lectures* (*2*,*5 h*): Physical activity, child dental health screening and the International Child Development Programme.	**Nov 2010**	**4**. *Lectures* (*3 h*): Overweight in pregnancy, dental health, and general information.
	*Interactive lecture* (*1*,*2 h*): Social services’ role.		*Group discussions* (*1*,*5 h*): What has happened? and ‘from knowledge to praxis’.
	*Group discussions* (*1*,*5 h*): What has happened and what do we do next?		
	**Survey 2(20 min)**		**Survey 2(20 min)**
**Nov 2010**	**Survey 1(post**-**implementation)**	**April 2011**	**Survey 1(post**-**implementation)**

^1^ All seminars were free of charge and included morning and afternoon tea, lunch and a short summary at the end of the day.

^2^ The survey was sent out to ANC and CHC in August 2009 and to dental services and open pre-schools in February 2010. The first seminar was planned to be held in October 2009, but was postponed with short notice because of the 2009 flu pandemic. The survey was already sent out to ANC and CHC when the decision to postpone the seminar was made.

### Data collection

#### Surveys

Two surveys were developed and used in the study. *Survey 1* included questions on health promotion practices, knowledge and attitudes in relation to lifestyle counselling, and extent of collaboration between sectors (23–27 questions depending on professional group). The questions were inspired by the Swedish version of The WHO Collaborative Study Questionnaire for GPs [18], but the questions were adjusted to fit the study setting and to reflect the intended goals of the Salut Programme (Table 2). The following domains were explored: 1) Respondents in ANC, CHC and dental services were asked how many of their 10 latest clients they had raised different lifestyle topics with (see Table 3 for sector-specific topics) and 2) to what extent they had used motivational interviewing (MI) [19] in lifestyle counselling. 3) Respondents in CHC were asked to what extent they offered EPDS-screening post partum [20] and 4) to what extent they invited fathers for a separate visit during the child’s first 18 months. 5) Respondents in open pre-schools were asked how often they offered outdoor activities, 6) activities encouraging physical activity in children, and 7) they were also asked to provide information on snacks and beverages provided at pre-schools (tick box question; Table 4). 8) Finally, all respondents were asked about the extent of collaboration with other sectors (each sector named). The questions had five response alternatives ranging from ‘no client’ to ‘all clients’ (Q1), from ‘never’ to ‘always’ (Q2-6), and from ‘not at all’ to ‘to a very large extent’ (Q8).

**Table 2:** **Västerbotten County Council**’**s public health vision**, **and goals of the Salut Programme**

**VISION AND GOALS**	
**Vision of the Västerbotten County Council**	
By 2020, the health and well-being of the population will be the best in the world.	
**Main goal for the Salut Programme**	
Good health is achieved by salutogenic interventions in collaboration with societal actors and the family with the child’s best in focus. Through systematic improvements, interventions are developed and implemented to promote satisfactory conditions during childhood, increased physical activity, and healthy eating habits.	
**The goals aim for**:	
Module I	
Expectant parents	✓ avoidance of maternal and fetal pregnancy complications related to maternal lifestyle
	✓ healthy maternal weight gain during pregnancy
	✓ a minimum of 30 minutes daily physical activity
	✓ regular meals
	✓ five fruits and vegetables a day
	✓ tooth-brushing twice a day with fluoride toothpaste
	✓ regular dental health care visits
	✓ parents are feeling prepared for their parental roles
	✓ pregnant women are living in relations free from intimate partner violence
	✓ pregnant women refrain from tobacco, alcohol and drug use
Module II	
Children 0–18 months with parents	✓ normal weight development for 18-month olds’
	✓ retain of pre-pregnancy weight
	✓ sufficient sleep (parents and children)
	✓ environments free from tobacco and drug use, and alcohol use is limited
	✓ a minimum of one hour daily physical activity (play) for children
	✓ a minimum of 30 minutes daily physical activity for parents
	✓ avoidance of TV-viewing and TV/computer games for children
	✓ six months exclusive breastfeeding, and thereafter partly continued for one year or longer
	✓ introduction of five fruits and vegetables a day for children
	✓ five fruits and vegetables a day for parents
	✓ regular meals for both parents and children
	✓ avoidance of discretionary foods for children
	✓ tooth-brushing twice a day with fluoride toothpaste (from the first tooth for the children)
	✓ regular dental health care visits
	✓ parents feel confident in their parent roles
	✓ satisfying parental-child attachment and interplay
	✓ women and children live free from men’s violence

**Table 3:** **Change in professionals**’ **practices of raising different lifestyle topics in encounters**^**1**^

	**Midwives**			**Child health nurses**			**Dental hygienists/dental nurses**		
	**Raising the topic with more than half or all pregnant women (%)**		**Wilcoxon Signed Rank Test**	**Raising the topic with more than half or all parents (%)**		**Wilcoxon Signed Rank Test**	**Raising the topic with more than half or all clients (%)**		**Wilcoxon Signed Rank Test**
**Topic**	Pre	Post	p-value	Pre	Post	p-value	Pre	Post	p-value
Psychosocial health	73	86	.048	47	64	.053	-	-	-
Men’s violence against women/children	59	82	.**010**	11	36	.**000**	-	-	-
Tobacco	91	96	.581	72	89	.059	79	84	.690
Alcohol	91	96	.414	64	81	.109	0	21	.026
Drugs	91	82	.269	19	36	.**004**	-	-	-
Weight/BMI	76	86	.107	11	13	.169	5	26	.**006**
Discretionary foods	59	41	.790	23	43	.**005**	47	100	.**001**
Regular meals	59	46	.941	34	55	.035	53	100	.**001**
Beverage intake	-	-	-	-	-	-	63	100	.**000**
5 fruits and vegetables a day	67	82	.191	21	53	.**000**	32	100	.**000**
30 min daily physical activity	59	68	.129	11	36	.**000**	-	-	-
Parenthood/parent relationships	77	77	.623	64	83	.107	-	-	-
Tooth brushing/dental health (child)	-	-	-	55	89	.**001**	-	-	-
Weight/BMI (child)	-	-	-	77	81	.805	-	-	-
5 fruits and vegetables a day (child)	-	-	-	45	66	.**002**	-	-	-
Regular meals (child)	-	-	-	83	85	.907	-	-	-
Discretionary foods (child)	-	-	-	55	68	.026	-	-	-
Physical activity (child)	-	-	-	72	87	.068	-	-	-
Psychosocial health (child)	-	-	-	79	87	.337	-	-	-

^1^Question: ‘Consider your 10 latest clients at your clinic (target modified by professional group). With how many of these have you raised each of these topics?’ Response alternatives: ‘no client’, ‘less than half of the clients’, ‘half of the clients’, ‘more than half of the clients’ and ‘all clients’.

Significant p-values (p≤.01) are presented in bold font.

**Table 4:** **Supply of snacks and beverages at open pre**-**schools pre**- **and post**- **implementation**^**1**^ (**n**=**20**)

**Type of snacks and beverages provided**	**Level of supply (%)**		
	**Pre**	**Post**	**p-value**
Fruit and vegetables	85	100	.250
Sandwiches	100	100	1.000
Cookies, biscuits and sweet buns	60	15	.**004**
Sweetened beverages	50	5	.**004**
Unsweetened beverages	45	70	.125

^1^Question: ‘What kind of snacks are usually provided at the pre-school?’ A tick box was provided for each alternative.

Significant p-values (p≤.01) are presented in bold font.

*Survey 2* included 16 open-ended questions on perceived implementation facilitators and barriers. The questions were guided by the framework of Grol and Wensing who propose factors to be identified at several levels of the healthcare system [12]. An initial question asked respondents to describe their workplace’s preconditions for Salut Programme implementation, and thereafter perceived implementation facilitators and barriers in relation to: the programme content and implementation process; the situation, routine and practices of work; the clients; the immediate organisational environment (colleagues, managers/directors, working climate etc.); collaborating actors; the organisational preconditions (structure, resources, administration etc.) and current activities in the society. The final question gave the respondents an opportunity to add other important aspects.

*Survey 1* was pre-tested by 12 professionals (three per sector) and *Survey 2* by three professionals outside the study areas, which resulted in minor linguistic adjustments.

#### Procedure

*Survey 1* included serial numbers to allow for pre- and post- implementation comparisons and was sent by mail to the respondent’s work address prior to their first encounter with the Salut Programme, and 4–6 months after programme implementation. Three reminders were used, two via mail (the second with a new survey form), and one via telephone. All respondents that returned a completed survey form received a movie ticket, two if they answered both the pre- and post surveys. *Survey 2* was administered at seminars 2 and 4 with respondents given 20 minutes to write their responses. It was anonymous, with respondents only being asked to report their profession, allowing for group comparisons only between time points. The timeline for data collection is illustrated in Table 1.

### Study sample

**Figure 1:**
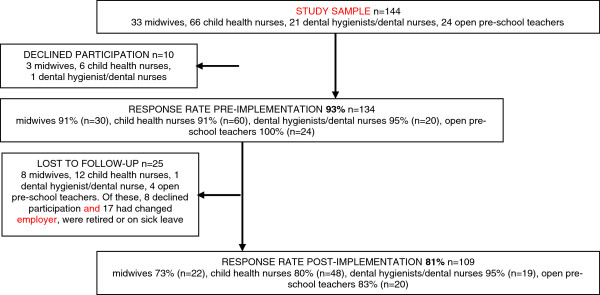
**Recruitment and response rates(Survey 1).**

The study sample (n=144) consisted at pre-implementation of midwives (n=33), child health nurses (n=66), dental hygienists/dental nurses (n=21) and open pre-school teachers (n=24). *Survey 1* was sent to all professionals in ANC and CHC in the 13 municipalities, and to all professionals in dental services clinics and open pre-schools that had agreed to participate in the Salut Programme. The response rate for *Survey 1* at pre-implementation was 93%, and 81% of those who replied, also responded post-implementation (Figure 1). Change of employer or retirement were the main reasons for loss to follow up. All professionals who attended learning seminar 2 (n=148) and 4 (n=116) were asked to complete *Survey 2*. The response rates for *Survey 2* were 96% (n=142) at seminar 2 and 85% (n=98) at seminar 4.

### Data analyses

Quantitative data from *Survey 1* were analysed with SPSS Statistics software (version 19). Descriptive statistics were calculated, and non-parametric techniques were used to evaluate significant changes from pre- to post- implementation as all outcome measures were based on ordinal and nominal data. The Wilcoxon Signed Rank Test was used to evaluate significant changes in health promoting practices and extent of collaboration between sectors and the McNemar Test was used to explore significant changes in supply of snacks and beverages at open pre-schools. Due to the requirements of both tests, professionals who did not respond both pre- and post- implementation were excluded from the analyses. To minimise the risk of false positive results due to multiple comparisons, statistical significance was defined as p≤.01.

Qualitative content analysis [21] was used to examine the free-text answers to the open-ended questions in *Survey 2*. Firstly, the answers from all professional groups were read through to get a sense of the whole and also the variation in responses. Secondly, facilitators and barriers were identified, listed, abstracted into categories, and these were then sorted according to the levels suggested by Grol and Wensing, i.e. the innovation, the individual professional, the client, and the social, organisational and the economic and political context [12,22]. The first author (KE) performed the initial analysis of all answers, and randomly selected sections were analysed by a co-author (EE). Any deviating interpretations were discussed and the categories slightly revised, resulting in a reassessment of free text answers. Finally, the number of respondents contributing to each category was counted and proportions reported. For conciseness, a decision was made to present the six most frequently described facilitators and barriers in each sector as these accounted for the majority of written responses.

### Ethics

The study was carried out in compliance with the ethical principles presented in the Helsinki Declaration. Ethics approval was obtained from the Ethics Review Board of Umeå University, Sweden (Ref. 08-168Ö).

## Results

### Sample characteristics

As shown in Table 5, the mean age ranged from 44 to 52 years (depending on professional group), all were females except two child health nurses, and the mean number of years in profession ranged from 8 to 19 years between different groups. The proportion of respondents working full time ranged from 44% (midwives) to 75% (dental hygienists/dental nurses). Most child health nurses (93%) had other work tasks than child health care, while 22% of midwives had other work tasks than antenatal care (Table 5).

**Table 5:** **Sample characteristics** (**n**=**134**)

**Professional group**	**Gender female**	**Age**	**Years in profession**	**Working full time**	**Other work tasks**
	**%**	**Mean** (**range**)	**Mean** (**range**)	**%**	**%**
Midwives (n=30)	100	52 (32–63)	15 (2–37)	44	22
Child health nurses (n=60)	97	52 (36–65)	14 (0–33)	52	93
Dental hygienists/dental nurses (n=20)	100	44 (23–60)	19 (2–40)	75	*
Open pre-school teachers (n=24)	100	49 (24–60)	8 (1–25)	50	*

* Only midwives and child health nurses were asked about other work tasks.

### Programme outcomes: changes in health promotion practices and collaboration (survey 1)

#### Health promotion practices in individual encounters

As shown in Table 3, significant improvements were found in several areas within CHC and dental services, and in one topic area, ‘men’s violence against women and children’ within ANC. Some areas showed a decrease, but this was not statistically significant. The percentages of midwives who reported raising different topics with ‘more than half or all pregnant women’ were already high before programme implementation. Child health nurses reported significantly improved practices for raising issues related to men’s violence against women and children, drug use, discretionary foods (adults), five fruits and vegetables a day (adults and children), 30 minutes daily physical activity and tooth brushing/dental health (children). Dental hygienists and dental nurses reported significant improvements in their practice for raising issues related to weight/BMI, discretionary foods, regular meals, beverage intake, and intake of five fruits and vegetables a day – representing major improvement in the majority of investigated areas in dental services.

#### Using motivational interviewing (MI) in lifestyle counselling

Child health nurses significantly improved their practice of using MI in lifestyle counselling (p=.006), with an increase from 51% to 60% reporting that they ‘often’ or ‘always’ used MI. Non- significant changes were found in ANC and dental services, with increases from 46% to 55% and from 61% to 67% for the two sectors respectively.

#### Inviting fathers to a CHC visit and screening mothers for postpartum depression (EPDS)

Child health nurses significantly improved their practices of inviting fathers for a separate visit during the child’s first 18 months (p=.000), with an increase from 0% to 38% reporting that they ‘often’ or ‘always’ invited fathers for a separate ‘father’s’ visit. There was a non-significant increase in EPDS-screening (p=.030), with 98% reporting that they ‘often’ or ‘always’ performed EPDS-screening post-implementation compared with 85% pre-implementation. However, unlike the EPDS-screening, inviting fathers for separate visits was a new work routine introduced as part of the programme.

#### Open pre-schools’ supply of healthy food and activities to encourage physical activity

There was a significant reduction in the reported supply of cookies, biscuits and sweet buns and sweetened beverages in open pre-schools (Table 4). However, no significant changes in activities to encourage children’s physical activity (p=.414) or outdoor activities (p=.107) were found. Only 15% of open pre-school teachers reported that outdoor activities were offered ‘often’ or ‘always’ at follow-up, an increase from 5%, while offering activities encouraging physical activity ‘often’ or ‘always’ decreased from 75% to 70%.

#### Collaboration between sectors

Table 6 shows that midwives significantly increased their collaboration with dental services, less so and not significantly with open pre-schools. Most midwives reported a high degree of collaboration with CHC both pre- and post- implementation. Child health nurses also significantly increased their collaboration with dental services. Most child health nurses reported a high degree of collaboration with ANC, consistent with the information reported by midwives. Dental hygienists and dental nurses significantly increased their collaboration with ANC and CHC, less so and not significantly with open pre-schools. Open pre-school teachers significantly increased their collaboration with dental services, with an increase from 10% to 60% reporting the collaboration to be considerable or very considerable.

**Table 6:** **Change in professionals**’ **collaboration between sectors**^**1**^

	**Collaborating sector**											
	**Antenatal care**			**Child health care**			**Dental services**			**Open pre-schools**		
	**Large or very large extent of collaboration (%)**	**Wilcoxon Signed Rank Test**		**Large or very large extent of collaboration (%)**	**Wilcoxon Signed Rank Test**		**Large or very large extent of collaboration (%)**	**Wilcoxon Signed Rank Test**		**Large or very large extent of collaboration (%)**	**Wilcoxon Signed Rank Test**	
**Professionals reporting collaboration**	Pre	Post	p-value	Pre	Post	p-value	Pre	Post	p-value	Pre	Post	p-value
Midwives	-	-	-	91	86	.132	5	41	.**000**	32	55	.019
Child health nurses	85	89	.059	-	-	-	31	76	.**000**	56	62	.298
Dental hygienists/dental nurses	19	63	.**000**	35	84	.**001**	-	-	-	5	37	.015
Open pre-school teachers	35	55	.032	55	70	.272	10	60	.**002**	-	-	-

^1^Question: ‘To what extent do you collaborate with professionals in following sectors?’ Response alternatives: ‘not at all’, ‘to a very small extent’, to a relatively small extent”, ‘to a large extent’ and ‘to a very large extent’.

Significant p-values (p≤.01) are presented in bold font.

### Perceived facilitators and barriers for programme implementation (Survey 2)

Perceptions about facilitators and barriers were largely shared across sectors, although some sector-specific factors were found. The six most frequently reported facilitators and barriers are presented for each professional group in Tables 7 and 8. The tables also provide information on changes in the frequency with which each factor was reported between the two occasions (Seminar 2 and 4).

**Table 7:** **The six most frequently reported *facilitators *for implementation in ANC**, **CHC**, **dental services and open pre**-**schools**

**Most frequently reported facilitators**	**Seminar 2**	**Seminar 4**	**Change**	**Level**
	**% (n)**	**% (n)**	**S2-S4**^**1**^	
***Antenatal care***	**N**^**2**^=**27**	**N**=**14**		
Collaboration with other sectors	33 (9)	57 (8)	➚	Social context
Support from work manuals and questionnaires	15 (4)	43 (6)	➚	Intervention
In line with existing work routines	37 (10)	21 (3)	➘	Interventions
Colleagues and working climate positive and supportive	37 (10)	14 (2)	➘	Social context
All professionals work the same way towards a shared goal	15 (4)	36 (5)	➚	Intervention
Managers positive/supportive	33 (9)	21 (3)	➘	Social context
***Child health care***	**N**=**48**	**N**=**37**		
Collaboration with other sectors	35 (17)	57 (21)	➚	Social context
Support from work manuals and questionnaires	23 (11)	51 (19)	➚	Intervention
Colleagues and working climate positive and supportive	40 (19)	35 (13)	➘	Social context
Programme clear or relevant	35 (17)	24 (9)	➘	Intervention
Parents willing to change lifestyles or are health conscious/informed	17 (8)	27 (10)	➚	Parents/clients
In line with existing work routines	17 (8)	22 (8)	➚	Intervention
***Dental services***	**N**=**19**	**N**=**16**		
Collaboration with other sectors	58 (11)	38 (6)	➘	Social context
Support from work manuals and questionnaires	47 (9)	19 (3)	➘	Intervention
Parents willing to change lifestyles or are health conscious/informed	16 (3)	44 (7)	➚	Parents/clients
In line with existing work routines	37 (7)	38 (6)	➚	Intervention
Colleagues and working climate positive and supportive	32 (6)	25 (4)	➘	Social context
Managers positive/supportive	26 (5)	25 (4)	➘	Social context
***Open pre***-***schools***	**N**=**15**	**N**=**15**		
Collaboration with other sectors	67 (10)	73 (11)	➚	Social context
Positive towards programme/interventions	40 (6)	53 (8)	➚	Professionals
In line with existing work routines	33 (5)	33 (5)	➔	Intervention
Colleagues and working climate positive and supportive	20 (3)	40 (6)	➚	Social context
Geographical proximity to collaborators	13 (2)	27 (4)	➚	Organisational context
Parents willing to change lifestyles or are health conscious/informed	27 (4)	13 (2)	➘	Parents/clients

^1^ ➚=Increase, ➘=decrease, ➔=no change

^2^ N=total number of professional in each professional group.

**Table 8:** **The six most frequently reported *barriers *for implementation in ANC**, **CHC**, **dental services and open pre**-**schools**

**Most frequently reported barriers**	**Seminar 2**	**Seminar 4**	**Change**	**Level**
	**% (n)**	**% (n)**	**S2**-**S4**^**1**^	
***Antenatal care***	**N**^**2**^=**27**	**N**=**14**		
Workload and shortage of time/staff/resources	89 (24)	57 (8)	➘	Organisational context
Geographical distance to collaborators	33 (9)	0	➘	Organisational context
Topics and questionnaires experienced as sensitive/intrusive or too extensive	19 (5)	50 (7)	➚	Parents/clients
Difficult to start or maintain collaborative relations	26 (7)	29 (4)	➚	Social context
Competing goals, demands and work tasks	22 (6)	14 (2)	➘	Professionals
Collaborative partners missing	22 (6)	7 (1)	➘	Social context
***Child health care***	**N**=**48**	**N**=**37**		
Workload and shortage of time/staff/resources	92 (44)	89 (33)	➘	Organisational context
Competing goals, demands and work tasks	58 (28)	41 (15)	➘	Professionals
Collaborative partners missing	15 (7)	46 (17)	➚	Social context
Insufficient managerial involvement/support (different levels)	27 (13)	30 (11)	➚	Social context
Geographical distance to collaborators	25 (12)	41 (15)	➚	Organisational context
Difficult to start or maintain collaborative relations	23 (11)	24 (9)	➚	Social context
***Dental services***	**N**=**19**	**N**=**16**		
Workload and shortage of time/staff/resources	89 (17)	31 (5)	➘	Organisational context
Lack of motivation/interest/time/capacity or resources (parents/clients)	53 (10)	25 (4)	➘	Parents/clients
Difficult to start or maintain collaborative relations	32 (6)	44 (7)	➚	Social context
Competing goals, demands and work tasks	26 (5)	19 (3)	➘	Professionals
Lack of or conflicting financial incentives	26 (5)	6 (1)	➘	Economic and political context
Insufficient managerial involvement/support (different levels)	16 (3)	25 (4)	➚	Social context
***Open pre***-***schools***	**N**=**15**	**N**=**15**		
Workload and shortage of time/staff/resources	60 (9)	73 (11)	➚	Organisational context
Insufficient work facilities of supplies	53 (8)	7 (1)	➘	Organisational context
Difficult to start or maintain collaborative relations	47 (7)	53 (8)	➚	Social context
Geographical distance to collaborators	40 (6)	20 (3)	➘	Organisational context
Insufficient managerial involvement/support (different levels)	13 (2)	47 (7)	➚	Social context
Lack of motivation/interest/time/capacity or resources (parents)	40 (6)	20 (3)	➘	Parents/clients

^1^ ➚=Increase, ➘=decrease

^2^ N=total number of professional in each professional group.

#### Facilitators

The pattern of reported facilitators differed from that of barriers as they were predominantly identified at the level of the intervention and the social context. The aspects of ‘*good collaboration with other sectors*’ were frequently reported, a factor that seemed to gain in importance by the end of the implementation process (Seminar 4) in ANC, CHC and open pre-schools. The importance of having *positive and supportive colleagues and working climate* was also reported as an important facilitator across sectors. All sectors reported the programme to be *in line with existing work routines*, and ANC, CHC, and dental services reported *good support from manuals and questionnaires*. Professionals in CHC, dental services and open pre-schools reported that *parents*’ *own willingness to change and health consciousness* was important. Having *positive and supportive managers* was among the main facilitators in ANC and dental services, although more frequently reported at Seminar 2. ANC also emphasised the importance of *professionals working in the same way towards a shared goal*. CHC perceived the *programme to be clear and relevant*. Open pre-school teachers stated that *being positive towards the programme*/*intervention* facilitated implementation. *Geographical proximity* was also reported among the main facilitators in open pre-schools. Interestingly, in all sectors organisational level and economic and political level facilitators were scarcely mentioned.

#### Barriers

*Workload and shortage of both time and staff* was the most frequently reported barrier, although the frequency with which this factor was reported decreased over time in all sectors except for open pre-schools. *Difficulties to start or maintain collaborative relations* were also a common barrier that seemed to increase in importance by the end of the implementation process (Seminar 4). Other main barriers reported were: c*ollaborative partners* were *missing* (ANC, CHC); *geographical distance to collaborators* (ANC, CHC, and open pre-schools); *insufficient managerial involvement and support* (CHC, dental services and open pre-schools). C*ompeting goals*, *demands and work tasks* posed a main barrier in ANC, CHC and dental services, although decreasing over time (in Survey I, 93% of the child health nurses reported having other work tasks than CHC (Table 5)). *Lack of motivation*, *interest*, *time or capacity*/*resources* among parents/clients were barriers experienced in dental services and open pre-schools, especially during the initiation phase (Seminar 2).

Some sector-specific barriers were found: The perception that *new topics and questionnaires could be perceived as sensitive*/*intrusive or too extensive* by expectant parents did seem to raise concern in ANC, especially by the end of the implementation process (Seminar 4) when this barrier was reported by half of the midwives. *Lack of or conflicting financial incentives* was reported in dental services, but mainly seemed to raise concern during the initiation phase (Seminar 2). *Insufficient work facilities and supplies* were mentioned as a barrier by almost half of the responding open pre-school teachers at Seminar 2. This was partly due to the fact that outdoor activities were suggested as one of the programme interventions, but many open pre-schools did not have sufficient or safe out-door environments to carry out these activities.

## Discussion

This study aimed to examine outcomes of a Swedish child health promotion programme on professionals’ self-reported health promotion practices, and to investigate perceived facilitators and barriers for programme implementation. The results show that self-reported health promotion practices and collaboration were improved to some extent in all sectors at follow up. Main facilitators for programme implementation included cross-sectoral collaboration and sector-specific work manuals/questionnaires for use as support in everyday practice. Main barriers included high workload, and shortage of time and staff. A strength of this study is that it spanned multiple sectors and included both outcome measures and cross sectional data on implementation facilitators and barriers, allowing for a deeper understanding of the context in which changes occurred. It is acknowledged that the outcome data are self-reported and thus susceptible to bias, so due caution is needed in interpreting the results.

The most significant changes were reported in CHC and dental services, and the least in ANC, the sector that may have the greatest opportunity to influence the target group. The results indicated that the majority of Salut activities were already in place within ANC. Midwives normally encounter a pregnant woman seven to ten times during pregnancy. The partners also seem more inclined to be involved in healthcare during pregnancy than after the child’s birth [5,23]. Thus, ANC might have needed more advanced interventions, especially since midwives have an excellent opportunity to involve both parents in health promotion activities. Interestingly, this was an issue raised by the midwives, as insufficient routines for involvement of fathers were reported as one of the barriers for programme implementation in ANC. A possible explanation for the relatively large change in dental services might be a novelty effect due to the introduction of new topics, or a simpler, less comprehensive intervention. The latter might explain why at the end of the implementation process (Seminar 4) fewer professionals in dental services reported support from structured work manuals as an important facilitator.

The framework used for analysing facilitators and barriers for change was valuable as it recognizes the importance of giving attention to different organisational levels [12]. During improvement efforts the focus is often on factors related to the individual professional [6] but as shown in this study, a number of factors can be involved and interact at multiple levels, many out of reach for the individual professional to influence.

Cross-sectoral collaboration emerged as one of the main facilitators for programme implementation. It is likely that the approach of gathering all sectors for seminars was successful in providing professionals with the opportunity to meet, interact and initiate or increase their collaboration, which in turn can create synergies and allow for a more integrated approach in provision of services [24,25]. Among the main facilitators identified were also the sector specific and structured work manuals and questionnaires. These results are consistent with findings from a previous study where the development of patient education toolkits and brochures was found to be one crucial component in improving nursing care in hospital and community settings [4].

The Salut Programme interventions were originally developed and tested within each profession in pilot areas before countywide implementation [16]. Thus, contextual factors that could impede change were to some extent already adjusted and accounted for [10,11,26,27]. A recent study of the implementation of a tool for lifestyle interventions in Swedish primary health care shows that a fixed implementation strategy (i.e. no tailoring of activities to meet professional needs) was not successful in terms of uptake of the intervention. Beliefs, values and behaviours linked to professional subcultures influenced the implementation, and most professional groups found the intervention more suitable for other professionals, contributing to a low level of adherence [28]. Thus, adapting approaches to different subgroups, preferably by involving end-users, is a strategy to enhance the likelihood of success [6,8,10,22], which is consistent with our findings.

Another commonly mentioned facilitator across sectors was that interventions were perceived to be in line with existing work routines. This is also an indication that the interventions were well adapted to the target context. However, an alternative interpretation is that the interventions did not introduce any novel practices or activities, possibly just a more structured way of working due to detailed work manuals and questionnaires. This might be one reason for the few significant changes in antenatal care. Since health promotion has a long tradition of being central in the Swedish antenatal care programme [29], midwives were already addressing most issues included in the programme pre-implementation, leaving little room for improvement.

The main barriers for programme implementation, high workload and shortage of time, staff or resources, are among the most well recognized in the implementation literature, and our results are thus consistent with previous research [30,31]. However, our findings suggest that improvements in health promotion practices and collaboration are possible to achieve, despite the presence of these common barriers. Even though positive outcomes were shown in this study, the characteristics of the barriers described indicate that there might have been a lack of overall organisational readiness for change, an important predictor of effective implementation, especially if changes are complex and the change process involves a great degree of interdependence [32]. It is plausible that outcomes in terms of health promotion practices and collaboration could have been further enhanced if attention had been given to readiness for change at the individual as well as the group and organisational levels. In addition, geographical distance was reported among the most important barriers in ANC, CHC and open pre-schools, a factor that should not be overlooked. Having shared premises has been shown to facilitate information transaction, communication and personal familiarity, while being located in separate buildings can constraint the team’s functioning and effectiveness [16,31,33]. Our results are consistent with these previous findings. As also indicated in the study, starting and maintaining new collaborative relations can be challenging, and this organisational network structure can be fragile due to a lack of shared management and due to demands both from both the teams and the organisations in which professionals are working [24]. Perceived barriers such as competing goals, demands and work tasks in ANC, CHC and dental services, or the conflicting financial incentives for performance in dental services were examples of such competing demands.

### Methodological considerations

The overall results of Survey I (outcome measure) have to be interpreted with caution, as findings from previous studies show that self-reported measures of adherence to guidelines often exceeds objective rates [34]. Nevertheless, the study validity is strengthened by high response rates (81% to 96%), this being well above what is commonly found in surveys with health care professionals [35]. Several factors known to enhance response rates were adopted in the study [36]. Furthermore, the first author and three of the co-authors participated in seminars and were introduced to all professionals. It is plausible that this familiarization further contributed to the overall high response rates. The risks of making Type I and Type II errors were considered in relation to the multiple comparisons made in the study, and a decreased p-value (p≤.01) for statistical significance was judged to be the most reasonable way to balance these risks.

Confounding factors cannot be readily ‘controlled’ for in implementation studies, as the context itself plays an important role in the level of success [7]. Thus, we do not know the extent to which sector specific continuous development and education contributed to programme outcomes. The results from the answers to the open-ended questions in *Survey 2* should also be interpreted with caution, as the extent of responses varied between respondents. However, the research team aimed to enhance the trustworthiness of the analysis by parallel coding and collaboration on categorization.

## Conclusions

This multisectoral programme for health promotion, based on sector-specific intervention packages developed and tested by end users, and introduced via interactive multisectoral seminars, shows potential for improving health promotion practices and collaboration across sectors. Consideration of the key facilitators and barriers for programme implementation as highlighted in this study can inform future improvement efforts.

## Competing interests

The authors declare that they have no competing interests.

## Authors’ contributions

KE, MN, RG, EE, IM and AI designed the study. KE collected the data, KE, EE, MN and ML conducted the analyses and KE drafted the manuscript in close collaboration with MN and RG. RS read and revised the manuscript with specific attention to language and clarity of presentation. All authors contributed in the writing process before the final version was approved. All authors read and approved the final manuscript.

## Pre-publication history

The pre-publication history for this paper can be accessed here:

http://www.biomedcentral.com/1471-2458/12/920/prepub

## References

[B1] Grol R Grimshaw J From best evidence to best practice: effective implementation of change in patients' care Lancet 2003 362 1225 1230 10.1016/S0140-6736(03)14546-1 14568747

[B2] Kerner J Rimer B Emmons K Introduction to the special section on dissemination: dissemination research and research dissemination: how can we close the gap? Health Psychol 2005 24 443 446 16162037 10.1037/0278-6133.24.5.443

[B3] Oldenburg B Glanz K Glanz K, Rimer B, Viswanath K Diffusion of Innovations Health Behavior and Health Education Theory, Research and Practice 2008 4 Jossey-Bass, San Francisco 313 333

[B4] Davies B Edwards N Ploeg J Virani T Insights about the process and impact of implementing nursing guidelines on delivery of care in hospitals and community settings BMC Health Serv Res 2008 8 29 10.1186/1472-6963-8-29 18241349 PMC2279128

[B5] Edvardsson K Ivarsson A Eurenius E Garvare R Nystrom ME Small R Mogren I Giving offspring a healthy start: parents' experiences of health promotion and lifestyle change during pregnancy and early parenthood BMC Public Health 2011 11 936 10.1186/1471-2458-11-936 22171644 PMC3282831

[B6] Grol RP Bosch MC Hulscher ME Eccles MP Wensing M Planning and studying improvement in patient care: the use of theoretical perspectives Milbank Q 2007 85 93 138 10.1111/j.1468-0009.2007.00478.x 17319808 PMC2690312

[B7] Greenhalgh T Robert G Bate P MacFarlane F Kyriakidou O Diffusion of Innovations in Health Service Organisations: A Systematic Literature Review 2008 Wiley, Chichester

[B8] Rogers E Diffusions of innovations 2003 5 Free Press, New York

[B9] Rogers EM Diffusion of preventive innovations Addict Behav 2002 27 989 993 10.1016/S0306-4603(02)00300-3 12369480

[B10] Harrison MB Legare F Graham ID Fervers B Adapting clinical practice guidelines to local context and assessing barriers to their use CMAJ 2010 182 78 84 10.1503/cmaj.081232 PMC2817341 19969563

[B11] Wensing M Bosch M Grol R Developing and selecting interventions for translating knowledge to action CMAJ 2009 182 85 88 10.1503/cmaj.081233 PMC2817342 20026633

[B12] Grol R Wensing M What drives change? Barriers to and incentives for achieving evidence-based practice Med J Aust 2004 180 57 60 15012583 10.5694/j.1326-5377.2004.tb05948.x

[B13] Lewin S Glenton C Oxman AD Use of qualitative methods alongside randomised controlled trials of complex healthcare interventions: methodological study BMJ 2009 339 b3496 10.1136/bmj.b3496 19744976 PMC2741564

[B14] Campbell M Fitzpatrick R Haines A Kinmonth AL Sandercock P Spiegelhalter D Tyrer P Framework for design and evaluation of complex interventions to improve health BMJ 2000 321 694 696 10.1136/bmj.321.7262.694 10987780 PMC1118564

[B15] Legare F Ratte S Gravel K Graham ID Barriers and facilitators to implementing shared decision-making in clinical practice: update of a systematic review of health professionals' perceptions Patient Educ Couns 2008 73 526 535 10.1016/j.pec.2008.07.018 18752915

[B16] Edvardsson K Garvare R Ivarsson A Eurenius E Mogren I Nystrom ME Sustainable practice change: Professionals' experiences with a multisectoral child health promotion programme in Sweden BMC Health Serv Res 2011 11 61 10.1186/1472-6963-11-61 21426583 PMC3077331

[B17] Eurenius E Lindkvist M Sundkvist M Ivarsson A Mogren I Maternal and paternal self-rated health and BMI in relation to lifestyle in early pregnancy - the Salut Programme in Sweden Scand J Public Health 2011 39 730 741 10.1177/1403494811418279 21930619

[B18] Geirsson M Bendtsen P Spak F Attitudes of Swedish general practitioners and nurses to working with lifestyle change, with special reference to alcohol consumption Alcohol Alcohol 2005 40 388 393 10.1093/alcalc/agh185 16043435

[B19] Rollnick S Miller WR Butler CC Motivational interviewing in health care: helping patients change behavior 2008 The Guilford Press, New York

[B20] Hewitt C Gilbody S Brealey S Paulden M Palmer S Mann R Green J Morrell J Barkham M Light K Methods to identify postnatal depression in primary care: an integrated evidence synthesis and value of information analysis Health Technol Assess 2009 13 1 145 147–230 19624978 10.3310/hta13360

[B21] Hsieh HF Shannon SE Three approaches to qualitative content analysis Qual Health Res 2005 15 1277 1288 10.1177/1049732305276687 16204405

[B22] Grol R Wensing M Eccles M Improving patient care. The implementation of change in clinical practice 2005 Elsevier, Oxford

[B23] New tools for parents - proposal for new forms of parental support [Nya verktyg för föräldrar - förslag till nya former av föräldrastöd] [In Swedish] http://www.fhi.se/PageFiles/3256/r200449nyaverktygforforaldrar.pdf

[B24] Axelsson R Axelsson SB Integration and collaboration in public health–a conceptual framework Int J Health Plann Manage 2006 21 75 88 10.1002/hpm.826 16604850

[B25] Zwarenstein M Goldman J Reeves S Interprofessional collaboration: effects of practice-based interventions on professional practice and healthcare outcomes Cochrane Database Syst Rev 2009 3 CD000072 19588316 10.1002/14651858.CD000072.pub2

[B26] Ovretveit J Understanding the conditions for improvement: research to discover which context influences affect improvement success BMJ Qual Saf 2011 20 18 23 10.1136/bmjqs.2010.045955 PMC3066695 21450764

[B27] Kaplan HC Brady PW Dritz MC Hooper DK Linam WM Froehle CM Margolis P The influence of context on quality improvement success in health care: a systematic review of the literature Milbank Q 2010 88 500 559 10.1111/j.1468-0009.2010.00611.x 21166868 PMC3037175

[B28] Carlfjord S Andersson A Lindberg M Experiences of the implementation of a tool for lifestyle intervention in primary health care: a qualitative study among managers and professional groups BMC Health Serv Res 2011 11 195 10.1186/1472-6963-11-195 21851596 PMC3170187

[B29] Swedish Association of Obstetrics and Gynecology [Svensk Förening för Obstetrik och Gynekologi] Maternal health care, sexual and reproductive health [Mödrahälsovård, sexuell och reproduktiv hälsa] [In Swedish] Report No 59 2008 Stockholm

[B30] Francke AL Smit MC de Veer AJ Mistiaen P Factors influencing the implementation of clinical guidelines for health care professionals: a systematic meta-review BMC Med Inform Decis Mak 2008 8 38 10.1186/1472-6947-8-38 18789150 PMC2551591

[B31] Robinson KL Driedger MS Elliott SJ Eyles J Understanding facilitators of and barriers to health promotion practice Health Promot Pract 2006 7 467 476 10.1177/1524839905278955 16885509

[B32] Weiner BJ A theory of organizational readiness for change Implement Sci 2009 4 67 10.1186/1748-5908-4-67 19840381 PMC2770024

[B33] Xyrichis A Lowton K What fosters or prevents interprofessional teamworking in primary and community care? a literature review Int J Nurs Stud 2008 45 140 153 10.1016/j.ijnurstu.2007.01.015 17383655

[B34] Adams AS Soumerai SB Lomas J Ross-Degnan D Evidence of self-report bias in assessing adherence to guidelines Int J Qual Health Care 1999 11 187 192 10.1093/intqhc/11.3.187 10435838

[B35] Cook JV Dickinson HO Eccles MP Response rates in postal surveys of healthcare professionals between 1996 and 2005: an observational study BMC Health Serv Res 2009 9 160 10.1186/1472-6963-9-160 19751504 PMC2758861

[B36] Field TS Cadoret CA Brown ML Ford M Greene SM Hill D Hornbrook MC Meenan RT White MJ Zapka JM Surveying physicians: do components of the "Total Design Approach" to optimizing survey response rates apply to physicians? Med Care 2002 40 596 605 10.1097/00005650-200207000-00006 12142775

